# Factors impacting the height of the interproximal papilla: A cross‐sectional study

**DOI:** 10.1002/cre2.728

**Published:** 2023-03-22

**Authors:** Hamdane Khaireddine, Tlili Mohamed, Rmida Arij, Khanfir Faten, Ben Amor Faten

**Affiliations:** ^1^ Research Laboratory in Oral Health and Oral‐facial Rehabilitation, Faculty of Dentistry of Monastir University of Monastir Monastir Tunisia; ^2^ Outpatients Department of Monastir Dental University clinic of Monastir Monastir Tunisia

**Keywords:** crown shape, interproximal papilla, papilla height, periodontal phenotype

## Abstract

**Objectives:**

To determine the relationship between different parameters (age, periodontal phenotype, contact point height, and crown shape) and the height of the interproximal papilla around the teeth of/in the maxillary anterior sector.

**Material and Methods:**

A total of 45 subjects were involved in this study, with 315 interproximal papillae of the maxillary anterior sector. The interproximal papillae were clinically classified according to the Norland and Tarnow classification. The periodontal phenotype was assessed by periodontal probe transparency through the marginal gingiva. The height of the papilla, the height of the contact points, and the width/length ratio of the crown were also measured using the periodontal probe. The relationship between the variables was studied using Pearson's correlation. Statistical significance was set at a value of *p* < .05.

**Results:**

A positive correlation was found between age and the papilla score. However, a negative correlation was noted between age and papilla height, with statistically significant values. A negative correlation was found between the papilla score and the rest of the studied clinical parameters. However, this correlation was not found with regard to the height of the papilla with the same parameters, except for the height of the contact points.

**Conclusions:**

A statistically significant relationship was noted between the appearance of the interproximal papillae and all the parameters studied.

## 1 INTRODUCTION

Dentists are currently faced with esthetic standards requiring a perfect balance between the pink of the gums and the white of the teeth. An incomplete interproximal papilla leads to the appearance of a black triangle, creating functional problems during phonation and biological problems due to the retention of bacterial plaque.

For modern dentistry respecting the esthetic imperatives, the interproximal papilla should be taken into account during the various restorative and surgical therapeutics. Fundamental knowledge of the different factors influencing the presence of this papilla is therefore necessary to respect its integrity.

Several authors have proposed different classification systems to assess the degree of papillary height loss. In 1997, Jemt et al. created a classification to evaluate the volume of interproximal papillae adjacent to single implant‐supported prostheses. Their objective was to provide a clinical assessment of the degree of recession and regeneration of the papillae adjacent to the restoration compared to that of the adjacent teeth. This index includes five different scores, ranging from total absence of the papilla to papilla hypertrophy (Jemt, 1997).

The following year, Norland and Tarnow proposed to classify the papillae into four classes based on three anatomical reference points: the contact point, the interproximal cementoenamel junction, and the facial cementoenamel junction. These authors excluded hypertrophic papilla to focus on papillary recession (Nordland & Tarnow, 1998).

In 2004, Cardaropoli et al. introduced an index evaluating the papillary height in relation to the contact point and the cementoenamel junction. It is the papillary presence index. This index allows estimating the height of the interproximal papilla even in the case of diastema. Follow‐up can therefore be undertaken during orthodontic treatment (Cardaropoli et al., 2004).

Many factors influencing the presence of the interproximal papilla have been studied. The distance between the base of the contact point and the top of the bony ridge is the best‐known factor, as it has been extensively documented by Tarnow et al. (1992) The majority of the studies on this parameter concluded that when the distance between the contact point and the bony ridge is less than or equal to 5 mm, the interproximal papilla is present in almost all cases. However, when the distance is greater than 7 mm, it is usually absent (Cho et al., 2006; Choquet et al., 2001; A. P. Kolte et al., 2014; de Lemos et al., 2013; Roccuzzo et al., 2018; Tarnow et al., 1992; Wu et al., 2003).

The impact of the clinical parameters on the presence of the interproximal papilla has been less studied in the literature compared to that of the radiological parameters (Chang, 2007; Chen et al., 2009; Chow et al., 2010; Fischer et al., 2014; Joshi et al., 2017; A. Kolte et al., 2016). In a study including 96 subjects, Cho et al. investigated the influence of different clinical parameters (crown shape, contact point height, periodontal phenotype) on the presence of interproximal papilla (Chow et al., 2010).

Kolt et al. studied the influence of both the crown shape and the interproximal space on the presence of the papilla. They reported a strong correlation between these two parameters (A. Kolte et al., 2016).

Joshi et al. also studied the impact of certain clinical and radiological parameters on papilla height. They concluded that the appearance of the papilla is significantly associated with the tooth shape, crestal bone height, gingival thickness, and gingival angle (Joshi et al., 2017).

Given the lack of data on a possible relationship between certain clinical parameters and interproximal papillae and given the importance of the presence of such parameters in terms of esthetics and function, this study proposed to determine the relationship between these parameters (age, periodontal phenotype, contact point height, crown shape) and interproximal papilla height in maxillary anterior teeth. The null hypothesis is that the presence of interproximal papilla is not directly impacted by age (1), periodontal phenotype (2), contact point height (3), and dental crown shape (crown width/length ratio) (4).

## 2 MATERIALS AND METHODS

The subjects examined for this study were recruited from the outpatient department at the University Dental Clinic of Monastir, Tunisia. The sample included patients, interns, dental residents, and staff of the above‐mentioned clinic.

The subjects included were adults (over 18 years of age) in good general health with intact anterior dentition (presence of teeth 14−24). They had no pathologies or medication influencing the periodontium, periodontitis, or history of periodontitis. Subjects with any of the following criteria were excluded from the study: gingival hypertrophy, previous or undergoing orthodontic treatment, diastemas, caries or proximal restorations, and signs of high attrition of the incisal edges.

The interview and all clinical assessments were performed by one calibrated examiner. For calibration before the study, the examiner performed repeated measurements of five subjects.

**Figure 1:**
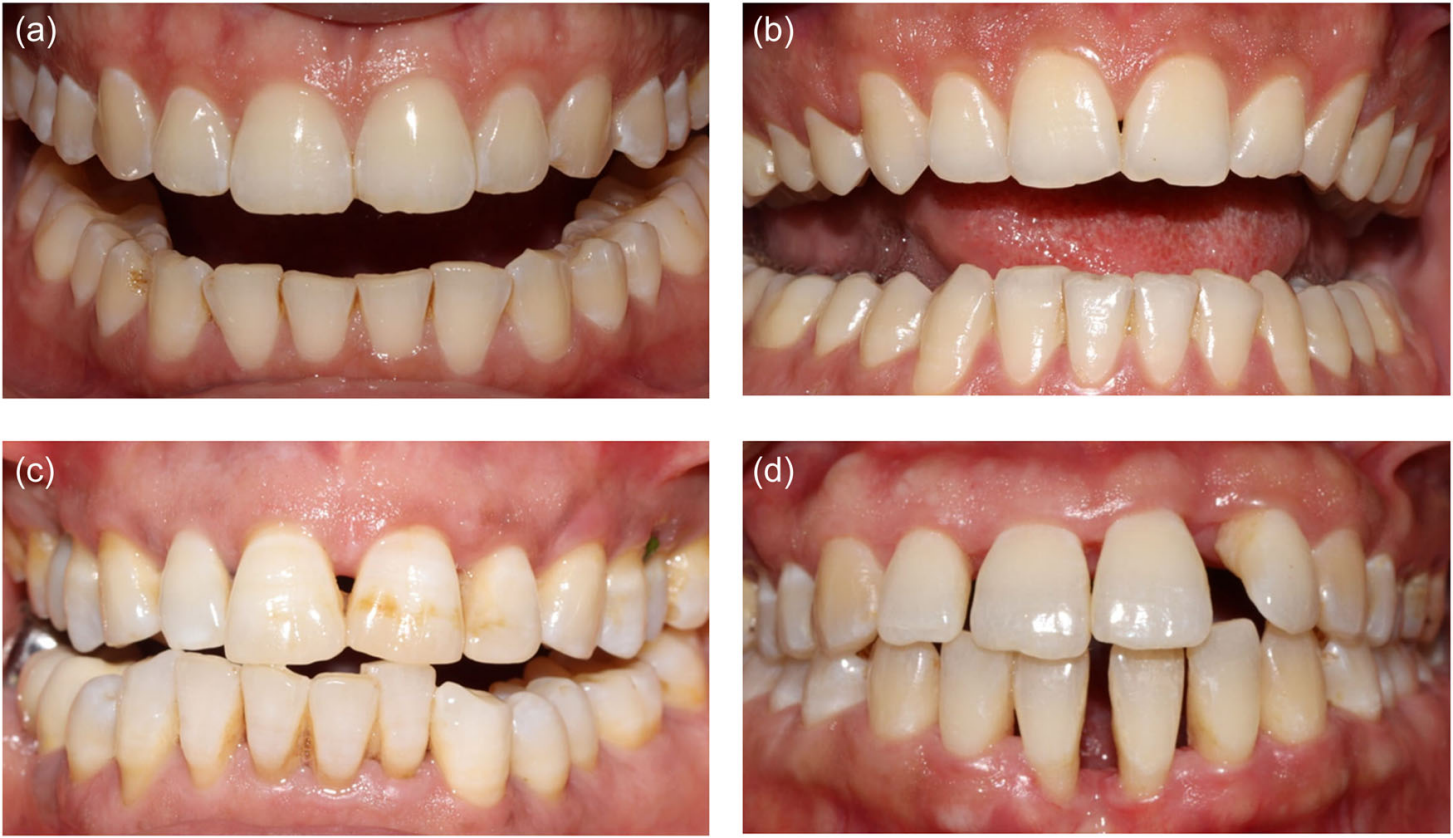
The different classes of papillae according to the classification of Norland and Tarnow. (a) Papilla between 11 and 21: Class 0; (b) papilla between 11 and 21: Class 1; (c) papilla between 11 and 21: Class 2; (d) papilla between 41 and 31: Class 3.

The interproximal papillae were clinically classified according to the Norland and Tarnow classification (Figure 1), with a score ranging from 0 to 3:

– Score 0: The interproximal papilla occupies the entire embrasure up to the most apical point of the contact point or surface.
– Score 1: The top of the papilla is located between the contact point and the most coronal point of the interproximal enamel‐cement junction (ECJ).
– Score 2: The apex of the papilla is located at or apical to the interproximal ECJ but coronal to the most apical point of the facial ECJ.
– Score 3: The papilla is located at the same level or apically to the most apical point of the facial ECJ.

The periodontal phenotype was assessed by the transparency of the periodontal probe (CPU 15 UNC; Hu‐Friedys) through the marginal gingiva and was classified as thick periodontium or thin periodontium (Malpartida‐Carrillo et al., 2021; De Rouck et al., 2009). A score of 0 was assigned for the thin periodontal phenotype and a score of 1 for the thick phenotype.

The height of the measured papilla represents the distance from the apex of the papilla to the line joining the gingival zeniths of the two adjacent teeth.

**Figure 2:**
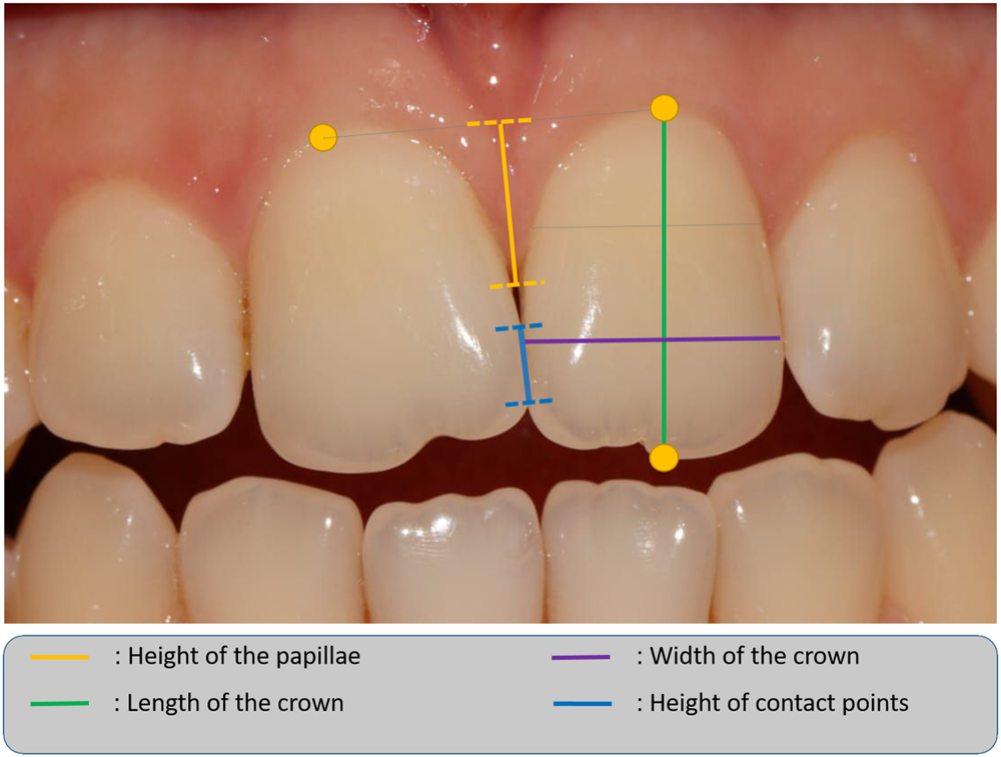
Illustration of the different clinical measurements performed.

The width‐to‐length ratio of the teeth bordering the papilla was measured by considering the length as the distance between the incisal edge or cuspidian tip and the gingival zenith. For width, it was measured at the incisal/middle third junction of the crown (Figure 2).

Data were analyzed using the Statistical Package for Social Science (IBM Corp. Released 2021; IBM SPSS Statistics for Windows; Version 26.0.: IBM Corp.). The relationship between the different parameters (age, periodontal phenotype, contact point height, and crown shape) and PSs/heights (papilla scores and height) was studied using Pearson's correlation. A negative value (negative correlation) is indicated when a clinical variable increases and the papillary height or papillary score decreases; however, a positive value (positive correlation) indicates that the two variables vary together in the same direction. Statistical significance was set at a value of *p* < .05.

## 3 RESULTS

Subject recruitment was performed between February and June 2022. The demographic characteristics are summarized in Table 1. A total of 45 subjects were included in the study out of 82 examined with a total of 315 interproximal papillae. They were from a Tunisian population. The subjects' average age was 29.75 years (±6.79; range 23−49). They all had good oral hygiene, with no evidence of active periodontal disease or history of periodontal disease. The frequencies of the different interproximal PSs are summarized in Table 2. The most frequent score according to the Norland and Tarnow classification was score 0, with the papilla completely occupying the interproximal space. No score higher than 2 was noted.

**Table 1:** Demographic characteristics and the mean height of the interproximal papilla of the study population.

Age, mean ± SD (range), year	29.75 ± 6.79 (23−49)
Sex, *n* (%)	
Male	21/45 (46)
Female	24/45 (54)
Papilla height, mean	3.3

*Note*: The height of the papilla is expressed in mm.

**Table 2:** Frequency of the different scores.

Papilla score	Score 0	Score 1	Score 2	Score 3
Percentages	78.4	20.6	1	0

*Note*: The different scores were assigned according to the classification of Norland and Tarnow.

The distribution of recessions (score greater than or equal to 1) according to location is summarized in Table 3. The highest prevalence was noted at the inter‐incisal papilla.

**Table 3:** Frequency of the different scores according to the location of the interproximal papilla.

	Score 0	Score 1	Score 2	Score 3
11−21	60%	40%	0%	0%
11−12	66%	26%	7%	0%
12−13	73%	27%	0%	0%
13−14	95%	5%	0%	0%
21−22	73%	27%	0%	0%
22−23	80%	20%	0%	0%
23−24	100%	0%	0%	0%

*Note*: The different scores were assigned according to the classification of Norland and Tarnow.

Pearson's correlations between the interproximal papilla measurements and the different clinical parameters are summarized in Table 4.

**Table 4:** Pearson's correlation between interproximal papilla measurements and different clinical parameters.

	Age	HCP	PP	W/H	
				Mesial	Distal
PS	0.324***	−0.291***	−0.196***	−0.276***	−0.434***
HP	−0.163**	−0.142**	0.39	−0.064	−0.119

*Note*: “*“ indicates statistical significance: *p ≈ .05; **p < .05; ***p < .001.

Abbreviations: HCP, height of contact points; HP, height of the papilla; PP, periodontal phenotype; PS, papilla score; W/H, width/height of the crown.

A positive correlation was found between age and papilla score and a negative correlation between age and papilla height, with statistically significant values (*p* < .5). With age, the papilla score increased and the papilla height decreased.

A negative correlation with statistically significant values was found between the papilla score and the rest of the clinical parameters studied (contact point height, periodontal phenotype, and width/height ratio of mesial and distal dental crowns). However, this correlation was not found for the height of the papilla with the same parameters, except for the height of the contact points.

## 4 DISCUSSION

In the present study, a correlation was noted between papilla appearance and age. The first null hypothesis was therefore rejected.

These results are in agreement with those found in the literature. In a study by Chang et al., 330 inter‐incisor papillae were visually assessed to study the impact of certain factors on their presence. A statistically positive relationship was found between inter‐incisal papilla recession and age, and a statistically negative relationship was found between age and papilla height in all the study groups (Chang, 2007).

In another study by Chow et al. investigating the impact of some parameters on the interproximal papilla, 96 subjects were divided into three age groups: <35 years (74 subjects), 35−49 years (11 subjects), and >50 years (11 subjects). Regardless of the interproximal site, the older group was found to have a higher incidence of papillary recession compared to the younger group (Chow et al., 2010).

Similar to periodontal disease, aging is not directly related to the loss of attachment at the interproximal papillae. However, the accumulation of certain factors over time can lead to recessions at this level. Age, therefore, increases the risk of recession.

It was found that a negative correlation exists between the rest of the clinical parameters studied (contact point height, periodontal phenotype, and width/height ratio of mesial and distal dental crowns) and the appearance of the interproximal papilla. The three null hypotheses were therefore rejected.

These results are in agreement with those reported in the literature.

In 2009, Chen et al. analyzed the impact of certain factors on the complete presence of interproximal papillae in a study including 102 interproximal sites at the anterosuperior sectors. They concluded that the thickness of the keratinized tissue is the main factor affecting the height of the interproximal papilla (Chen et al., 2009).

In the previously cited study conducted by Chow et al., the authors concluded that papilla appearance is significantly associated with the subject's age, tooth shape, interproximal contact length, alveolar bone height, and interproximal gingival thickness (Chow et al., 2010).

In a more recent study by Joshi et al., 150 interproximal papillae in 30 patients were evaluated using clinical and study models to investigate the correlation with certain factors. The appearance of interproximal papillae was reported to be significantly associated with tooth shape, alveolar bone height, and gingival thickness (Joshi et al., 2017).

**Figure 3:**
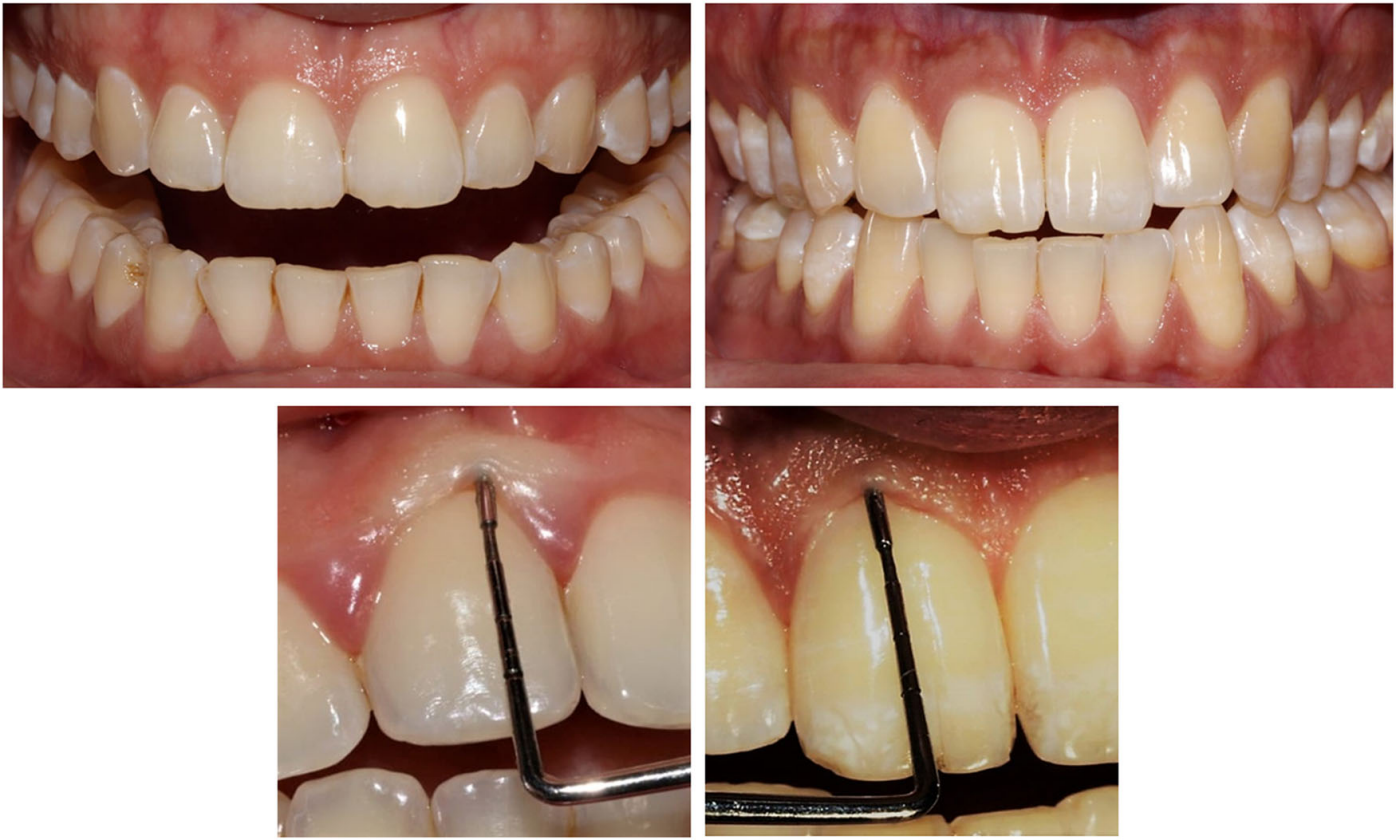
Different periodontal phenotypes and coronal morphologies: the photograph on the right represents a thin phenotype with an oval crown and that on the left represents a thick periodontal phenotype with a square coronal shape. We notice the whitening of the gingiva following the insertion of the periodontal probe in the thick periodontal phenotype.

However, some of these parameters (periodontal phenotype and dental crown width/length ratio) are only significantly correlated with PSs and not with papilla height. The fact that a thicker periodontal phenotype is not correlated with higher papillary height seems to be quite consistent. This type of periodontium has less scalloped gingiva with shorter papillae, which counterbalances the lower prevalence of attachment loss at this level. Similarly, for the width/length ratio of dental crowns, a square‐shaped crown is associated with a lower interproximal papilla compared to an oval‐shaped crown (Figure 3).

In addition to the parameters already mentioned, a positive correlation between the height of the interproximal papilla and the gingival angle is reported in the literature. This angle is formed by the intersection of the two lines joining the most coronal points of the interproximal papillae to the gingival zenith. This angle is used with the papillae height to assess the gingival contour (Joshi et al., 2017). A positive correlation is also reported between papilla height and interdental width (Chen et al., 2009; A. Kolte et al., 2016).

The results of this work showed that the interproximal papilla is an anatomical structure, which, under certain constraints and under the influence of certain factors, can lose its height and give the appearance of an unsightly black triangle which can be badly experienced by the patient. These results are consistent with some findings reported in the literature and are inconsistent with others with regard to the influence of certain factors on the papilla. However, to date, no systematic review or meta‐analysis has been conducted to discuss this topic and confirm the influence of these factors. This research could therefore contribute to the elaboration of a study with a higher level of scientific evidence. In addition, the significant correlation found in this work between the appearance of the papilla and some predisposing factors (periodontal phenotype, shape of the crown, and height of the contact points) allows us to better understand, to manipulate these parameters for preventive purposes, or to manage the loss of interproximal attachments. Indeed, mucogingival surgery can be performed to thicken the keratinized tissues in case of a thin periodontal phenotype. Prosthetic design and restorative dentistry can modify the shape of the crown and the height of the contact points to obtain an environment favorable to the stability or regeneration of the interproximal papilla.

In the subjects examined in the present study, the papillae with a score of 2 or 3 were almost absent (1% of the papillae for score 2 and 0% for score 3), which can be explained by the strict exclusion criteria applied in this study (Table 2). These criteria included the presence of active periodontal disease or a history of periodontal disease, which is the primary cause of loss of attachment in interproximal papillae.

The results of this study also showed that inter‐incisal papillae had the highest prevalence of recessions, with 40% of the papillae affected by recessions (Table 3). This can be explained by the presence of a maxillary labial frenulum at this level, which can present a low insertion favoring plaque accumulation and loss of attachment.

The present study presents some limitations. The method applied to measure distances (height of papillae, height of contact points, and crown width/height ratio) may be imprecise due to the limited graduations of the periodontal probes. In addition, the measurements were not taken from flat surfaces. They were taken from three‐dimensional structures with convexities and concavities, which may have distorted the reading of the results on the periodontal probe.

Similarly, the method used to evaluate the thickness of the keratinized gingiva is another limitation despite its simplicity and ease of reproducibility. Indeed, the reported results are thin or thick periodontium. Other methods, such as ultrasound, have been used in the literature to measure soft tissue thickness (Müller et al., 2000).

Future studies should therefore include standardized, simple, and reproducible measurement methods with a larger study population. The latter should include several ethnicities to assess and take into account interethnic differences in the interpretation of the results. Studies with a higher level of scientific evidence (systematic reviews and meta‐analyses) should therefore be conducted to confirm or contradict these results.

## 5 CONCLUSION

In the present study, a statistically significant relationship between the appearance of interproximal papillae and various parameters (age, periodontal phenotype, height of contact points, and shape of the crown) was found. Understanding these different factors is essential to prevent and manage tissue loss in the papillae to obtain optimal esthetic results from the various therapies performed.

## AUTHOR CONTRIBUTIONS

Dr Hamdane Khaireddine and Pr Ben Amor Faten conceived of the presented idea. Dr Hamdane Khaireddine, Dr Tlili Mohamed, and Doctor Rmida Arij developed the analytic methods. Dr Khanfir Faten and Pr Ben Amor Faten verified the analytic methods. Dr Khaireddine Hamdane has carried out the interview and all clinical assessments. Dr Tlili Mohamed took the photographs. All authors discussed the results and contributed to the final manuscript.

## CONFLICT OF INTEREST STATEMENT

The authors declare no conflict of interest.

## Data Availability

Data that support the findings of this study are available on request from the corresponding author. The data are not publicly available due to privacy.
